# Emerging Technologies in the Treatment of Orbital Floor Fractures: A Systematic Review

**DOI:** 10.3390/medicina61081330

**Published:** 2025-07-23

**Authors:** Lorena Helgers, Ilze Prikule, Girts Salms, Ieva Bagante

**Affiliations:** 1 Riga Stradins University, LV-1010 Riga, Latvia; 2 Centre of Oral and Maxillofacial Surgery, Pauls Stradins Clinical University Hospital, LV-1002 Riga, Latvia; 3 Institute of Stomatology, Riga Stradins University, LV-1007 Riga, Latvia; 4 Baltic Biomaterial Centre of Excellence, Headquarters at Riga Technical University, LV-1048, Riga, Latvia

**Keywords:** orbital floor fractures, virtual surgical planning, 3D printing, patient-specific implants, intraoperative navigation

## Abstract

*Background and Objectives*: Orbital floor fractures are challenging to treat, due to the complex orbital anatomy and limited surgical access. Emerging technologies—such as virtual surgical planning (VSP), 3D printing, patient-specific implants (PSIs), and intraoperative navigation—offer promising advancements to improve the surgical precision and clinical outcomes. This review systematically evaluates and synthesizes current technological modalities with respect to their accuracy, operative duration, cost-effectiveness, and postoperative functional outcomes. *Materials and Methods*: A systematic review was conducted according to the PRISMA 2020 guidelines. The PubMed, Scopus, and PRIMO databases were searched for clinical studies published between 2019 and September 2024. Out of 229 articles identified, 9 met the inclusion criteria and were analyzed using the PICO framework. *Results*: VSP and 3D printing enhanced diagnostics and presurgical planning, offering improved accuracy and reduced planning time. Pre-bent PSIs shaped on 3D models showed superior accuracy, lower operative times, and better cost efficiency compared to intraoperative mesh shaping. Custom-designed PSIs offered high precision and clinical benefit but required a longer production time. Intraoperative navigation improved implant positioning and reduced the complication rates, though a detailed cost analysis remains limited. *Conclusions*: VSP, 3D printing, and intraoperative navigation significantly improve surgical planning and outcomes in orbital floor reconstruction. Pre-bent PSIs provide a time- and cost-effective solution with strong clinical performance. While customized PSIs offer accuracy, they are less practical in time-sensitive settings. Navigation systems are promising tools that enhance outcomes and may serve as an alternative to custom implants when time or resources are limited.

## 1. Introduction

Orbital floor fractures often cause both functional and aesthetic problems, significantly impairing patients’ quality of life [1,2]. While the findings vary across studies, orbital floor fractures account for approximately 30–40% of facial fractures. Isolated orbital floor fractures constitute about 4–16% [3,4,5]. Their repair can be challenging due to the limited operative visibility and working field within the orbit’s complex anatomy [6,7,8]. Traditional methods rely on intraoperative freehand shaping and the surgeon’s experience [9]. Safe, fast, and accurate treatment is essential for a successful outcome [7,10].

Advanced surgical technologies such as orbital titanium meshes, 3D printing, preoperative planning software, and imaging tools offer the potential to improve surgical outcomes [6,7,11]. However, one might question whether these technologies are truly more efficient. Are the investments in innovation justified, or are traditional techniques sufficient for effective outcomes? This systematic literature review aims to analyze and compare recent advancements in orbital floor surgical reconstruction to answer this question.

The objectives of this research were as follows: to review the application of virtual surgical planning (VSP) and 3D printing to diagnostics and presurgical planning; to analyze whether PSIs enable a more accurate and time- and cost-efficient surgery with enhanced functional outcomes; and to evaluate the impact of intraoperative navigation on the surgical precision, efficiency, and postoperative outcomes.

## 2. Materials and Methods

### 2.1. Search Strategy

A comprehensive literature review adhering to the PRISMA 2020 checklist guidelines [12] was conducted in PubMed, Scopus, and PRIMO. Initially, a broad range of emerging technologies for the treatment of orbital floor fractures was considered; however, further narrowing was conducted to focus on selected technologies, as including all new technologies was beyond the scope of this review. For reference management software Endnote 21.4 (Philadelphia, PA, USA) was used. The search strategy was then modified, as shown in Table 1.

### 2.2. Eligibility Criteria

To qualify for inclusion in the review, studies needed to fulfil the strict requirements listed below. All studies failing to meet the inclusion criteria were excluded.

Inclusion Criteria:

- Articles published from 2019 to September 2024;
- Condition: only orbital floor or both orbital floor and medial wall affected;
- Condition: only primary fracture treatment;
- Language: English;
- Peer-reviewed publications;
- Clinical studies (retrospective or prospective).

Exclusion Criteria:

- Case reports;
- Preclinical laboratory or animal studies;
- Sample size < 10 fractures treated;
- Pediatric patients;
- Concomitant facial fractures (e.g., zygomaticomaxillary, nasal).

### 2.3. Study Selection

**Figure 1:**
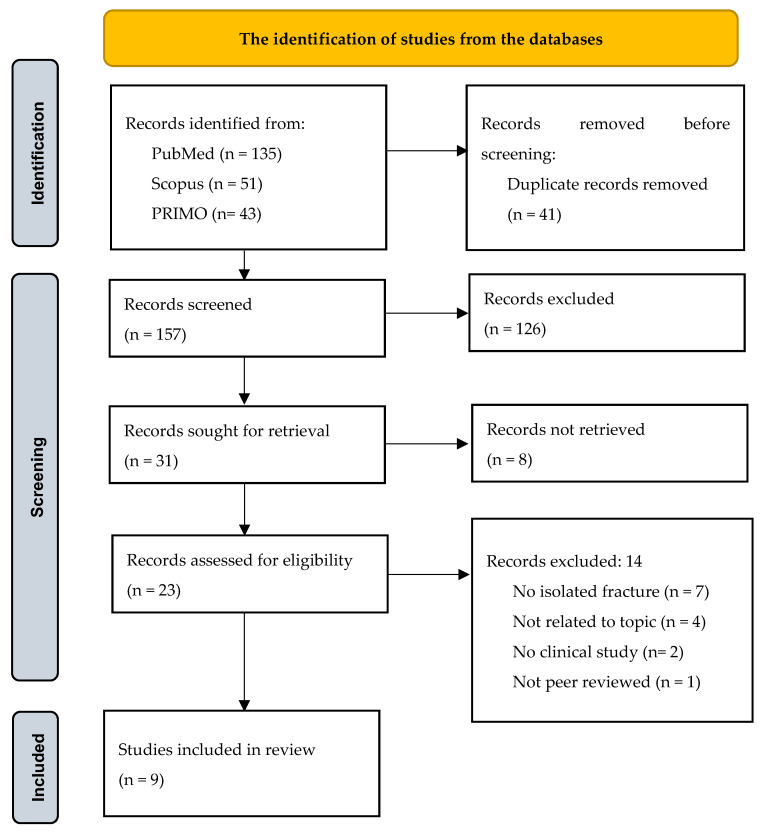
PRISMA flow diagram for the identification of studies from the databases.

The search yielded 229 articles. After duplicate removal (*n* = 41), 157 titles and abstracts were screened. Of these, 31 full-text articles were sought, and 9 studies met all eligibility criteria. Two reviewers independently conducted the selection process. Disagreements were resolved through discussion. No automation tools were used. The study selection process is depicted in the PRISMA flow diagram (Figure 1).

### 2.4. Data Extraction

Data from the 9 included studies were extracted using a standardized spreadsheet (Apple Numbers), based on the PICO framework:

(1) Author, year, journal;
(2) Study design;
(3) Sample size;
(4) Intervention;
(5) Comparison group;
(6) Outcome variables;
(7) Main conclusions.

A summary of these data is provided in Appendix A.

### 2.5. Risk of Bias Assessment

To assess the methodological quality of the included studies, we applied the ROBINS-I tool (Risk Of Bias In Non-randomized Studies of Interventions, the Cochrane UK charity No. 1045921 and a company No. 03044323) [13], as most included studies were retrospective. The tool evaluates seven domains of bias: 1—bias due to confounding; 2—bias in the selection of participants; 3—bias in the classification of interventions; 4—bias due to deviations from the intended interventions; 5—bias due to missing data; 6—bias in the measurement of outcomes; and 7—bias in the selection of the reported results. Each domain was graded as having a low, moderate, serious, or critical risk of bias. Two reviewers independently assessed each study, and discrepancies were resolved through consensus. A summary of the risk of bias ratings is presented in Table 2. One randomized controlled trial (Amin et al., 2024 [11]) was assessed using the Cochrane RoB 2.0 tool (The Cochrane UK charity No. 1045921 and a company No. 03044323) [14].

### 2.6. Support and Data Availability

The authors acknowledge financial support from the European Union’s Horizon 2020 research and innovation program under the grant agreement No. 857287 (BBCE – Baltic Biomaterials Centre of Excellence) and the authors declares no competing interests. The data extracted for this review are available upon request.

## 3. Results

### 3.1. Study Characteristics: Study Design and Population

A total of nine studies published between 2019 and September 2024 were analyzed in detail, having mostly retrospective designs, with one prospective randomized clinical trial [11]. They included 313 patients (ages 18+) who underwent primary surgery for an orbital floor or combined floor and medial wall fractures. The sample sizes ranged from 22 to 73 patients. Six studies compared the outcomes with a conventional group [6,8,11,15,16,17], while three studies lacked a control group [7,9,18]. Most retrospective studies showed a moderate risk of bias, primarily due to confounding and outcome measurement. No studies were excluded based on the risk of bias. One randomized controlled trial (Amin et al., 2024 [11]) was assessed using the Cochrane RoB 2.0 tool. It showed low a risk of bias across all domains. A more detailed synthesis of the results is presented below in Table 2 and in Appendix A.

### 3.2. Synthesis of Results: Intervention, Outcome Measures, and Author Conclusions

#### 3.2.1. Diagnostic Tools: Presurgical Virtual Planning and 3D Printing

Process: This process is based on imaging data mirrored from the contralateral side to produce computer-aided design (CAD)-based 3D virtual models, assisting diagnosis and surgical planning [7,15,18]. A 3D-printed replica aids the presurgical bending of a standard orbital floor mesh directly on the model, rather than solely during the surgery [8,11,17].

Findings: Several studies reported improved implant positioning accuracy, assessed through superimposition and 3D analysis, although VSP was mainly only used in the intervention groups [6,7,9,15,18]. They highlighted the practicability and efficiency of VSP, reducing the processing time to 15–20 min [6,17,18].

The studies also reported positive outcomes from using 3D-printed models for orbital reconstruction, highlighting benefits such as enhanced preoperative planning through precise fracture visualization and improved understanding of patient-specific anatomy [11,16,17]. Three-dimensional printing enabled the pre-shaping of orbital plates outside the operating room [11]. The studies found 3D printing to be cost-effective, as affordable in-house 3D printers are broadly available and can reduce surgery expenses by minimizing time in the operating room, therefore reducing the time of general anesthesia [16,17].

#### 3.2.2. Pre-Bending on a 3D Model

Standard mesh was pre-bent on a patient-specific 3D-printed model, rather than bent intraoperatively [11,16,17].

Accuracy: The studies found increased precision. Sigron et al. [16] reported smaller orbital volume (OV) pre- and postoperative differences in the PSI group (1.0 mL vs. 1.6 mL), with a statistically significant difference in the conventional group. Amin et al. [11] mentioned a better fit and predictable results in terms of the OV. The study by Sigron et al. [17] concluded that the use of pre-bent implants allowed for less invasive insertion by minimizing multiple fitting attempts and reducing the risk of implant misalignment.

Time- and cost-efficiency: Pre-bent implants significantly reduced the operating times by 20.7 min in the study by Amin et al. [11], 42.5 min in Sigron et al. [16], and 35.9 min in Sigron et al. [17]. The significant reduction in surgery duration ultimately led to notable cost savings in all three studies by minimizing the time spent in the operating room. For example, Sigron et al. [16] observed cost savings of up to USD 4377.50 in Switzerland, calculated by multiplying the time saved by the cost per minute.

Functional outcomes: Sigron et al. [17] reported improved functional and cosmetic outcomes with a pre-bent PSI compared to the intraoperatively adapted mesh, although the differences between the groups were not statistically significant. In the intervention group, 75.0% of preoperative diplopia and 77.8% of ocular motility impairment cases were resolved. In the conventional group, 71.4% of diplopia and 60.0% of motility impairments were resolved. Enophthalmos improved in both groups.

#### 3.2.3. Custom Fabrication

Customized CAM-designed patient-specific implants were manufactured through a computer-aided manufacturing (CAM) process, using additive manufacturing methods such as selective laser melting or 3D printing via an external manufacturer [6,9,15].

Accuracy: The accuracy was evaluated by comparing the pre- and postoperative implant positional deviations in all three studies, with additional comparison of the volume differences in the study of Consorti, Betti, et al. [6]. Krasovsky et al. [15] found that PSIs achieved significantly superior accuracy related to the planned position (0.58 mm average deviation) compared to traditional mesh techniques (1.54 mm). Probst et al. [9] also noted reliable reconstruction accuracy using freehand-placed customized PSIs. In contrast, Consorti, Betti, et al. [6] found comparable accuracy between custom-fabricated PSIs and 3D preformed stock implants using VSP and intraoperative navigation for both groups, with minimal deviations (0.62 mm vs. 0.69 mm).

Time- and cost-efficiency: Krasovsky et al. [15] reported a 14% increase in surgery time for the PSI group, which they noted was due to the implementation of a new technique. The overall time of the PSI virtual planning and 3D printing in their study took 3 days: 1 day for segmentation and design, and 2 days for 3D printing and external manufacturing. Probst et al. [9] reported an average operation time of 87 min. Consorti, Betti, et al. [6] found 3D preformed implants potentially more time- and cost-efficient, as the overall time of cutting the preformed implants intraoperatively only took about 1 min.

Functional outcomes: Krasovsky et al. [15] found that patients treated with PSIs experienced significantly better outcomes and fewer immediate and long-term complications, such as diplopia (25% vs. 55%) and restricted eye movement (8% vs. 36%), compared to those treated with prefabricated implants. Consorti, Betti, et al. [6] also reported favorable outcomes but with no significant difference for both PSI and preformed implants, with both groups achieving full diplopia resolution and 92.3% success in enophthalmos correction. Probst et al. [9] found no relationship between the implant positioning accuracy and unfavorable functional outcomes related to diplopia.

#### 3.2.4. Intraoperative Navigation

Intraoperative navigation combines VSP with real-time image-guided feedback to achieve precise implant placement. Preoperative CT data and implant STL files were loaded into navigation software, enabling a magnetic or infrared-based system to overlay the patient’s anatomy with the planned implant position during surgery [6,7,8,18].

Accuracy: The accuracy was evaluated according to the postoperative placement compared to the preoperative implant planning or the orbital volume (OV) difference. All studies observed an enhancement in the implant positioning accuracy. Gallego-Albertos et al. [8] and Consorti, Monarchi, et al. [18] found that navigation-assisted surgeries significantly improved the control over the plate positioning, particularly in the posterior intact bony ledge of the orbit, leading to a reduced need for revision surgeries. Consorti, Monarchi, et al. [18] reported an average deviation of 0.692 mm between the final plate position and the originally planned position. Raveggi et al. [7] also noticed significant enhancements in plate alignment. Gallego-Albertos et al. [8] additionally observed a higher volume reduction in the navigation group; however, the deviation was not significant. Consorti, Betti, et al. [6] demonstrated no significant difference in accuracy between the two implant types, attributing comparable outcomes to the precise control offered by navigation.

Time- and cost-efficiency: Consorti, Monarchi, et al. [18] stated there were advantages in terms of time and cost savings. The other studies did not discuss the surgery times and costs.

Functional outcomes: Navigation significantly improved the functional outcomes across the studies, reducing diplopia, enophthalmos, and re-interventions [7,8,18]. Only 3 out of 73 patients needed revision surgery in the study of Raveggi et al. [7]. Additionally, Gallego-Albertos et al. [8] noted significant reductions in abnormal globe positioning and faster ocular motility recovery. Consorti, Monarchi, et al. [18] further noted minimized complications, such as repeated implant fittings or soft tissue damage around the periorbital area.

## 4. Discussion

Emerging technologies play a vital role in orbital floor fracture treatment as time and economic efficiency, along with practicability in the clinical workflow, are indispensable. Overall, this review presents advancements in orbital floor repair using VSP, 3D printing, PSIs, and intraoperative navigation. These technologies have been evaluated in terms of their improved accuracy, reduced surgical time, cost-efficiency, and improvements in functional outcomes.

This review is strengthened by its comprehensive search methodology and selection of recent studies. However, the narrow focus on orbital floor fractures consequently led to a relatively small number of studies for the analysis. Additionally, significant heterogeneity was present across the included studies, particularly in the study design, intervention modality, and outcome reporting.

Of the nine included studies, eight were retrospective in nature [6,7,8,9,15,16,17,18], and only one was a prospective randomized controlled trial [11]. This imbalance introduces a moderate risk of bias across the dataset, as retrospective studies are more susceptible to selection bias, uncontrolled confounding, and inconsistent data collection. The lack of blinding and randomization in most studies may also have affected the objectivity of the outcome assessment.

Moreover, the studies used varied types of implants: some evaluated pre-bent standard titanium meshes shaped on 3D-printed models, others analyzed customized patient-specific implants (PSIs) manufactured using CAM technologies, and still others used 3D preformed stock implants. In many cases, the implant types were combined with additional technologies such as intraoperative navigation or VSP, making it challenging to isolate the effect of a single intervention. For example, navigation-assisted reconstruction using a preformed implant may offer similar accuracy to a customized PSI without navigation, but this comparison was not uniform across studies.

Different outcome measures further limited a comparative analysis, as conclusions had to rely on the findings of only one or two studies. Additionally, two studies shared similar patient populations, which could lead to repeated results that may not independently validate the findings. The overlap of authors in this review also limits the diversity, as their studies could share similar biases or perspectives. Independent studies from various clinical settings would be more valuable to validate the findings.

The results demonstrate that VSP and 3D printing are essential time- and cost-effective tools for diagnostics and surgery planning, enhancing the visibility of each patient’s unique anatomy and enabling pre-shaped implants. Consorti, Betti, et al. [6] additionally showed the potential of VSP in optimizing traditional implant planning by virtually designing the intraoperative freehand removal of excess mesh.

Pre-bent PSIs outperformed in precision and efficiency, reducing the demand for multiple fitting attempts and enabling a less invasive approach, which may have contributed to the faster recovery and fewer complications. The surgery time was significantly reduced (by 20.7 to 42.5 min), which lowered the anesthesia duration and its associated risk, as well as the costs, making pre-bent PSIs both clinically and economically viable. However, Sigron et al. [16] emphasized that the costs differ markedly depending on the staffing levels, infrastructure expenses, and healthcare systems across countries.

Customized implants also showed high accuracy (average 0.58 mm displacement by Krasovsky et al. [15]) and good functional outcomes. However, the study by Consorti, Betti, et al. [6] demonstrated that intraoperative navigation can match customized implant precision with preformed implants (0.69 mm deviation). Additionally, the time efficiency was insufficient (e.g., 3 days by Krasovsky et al. [15]); hence, preformed or pre-bent options are faster and more cost-effective.

The use of intraoperative navigation should be promoted, as it provides further precision, minimizes complications, and offers real-time feedback, saving time and cost, although detailed data are lacking.

Despite clear clinical benefits, formal cost-effectiveness analyses of VSP, 3D printing, and navigation are lacking. Most studies report time savings but omit a structured economic evaluation. Furthermore, the predominance of single-center retrospective studies with small sample sizes restricts the generalizability.

The results between studies may vary, as the success of a technology could be evaluated differently depending on the measured outcomes and their interpretations. Functional outcomes and time- and cost-efficiency were not always considered in the studies, which complicates comparisons across studies. While the accuracy was often assessed through positional differences compared to presurgical planning, other studies evaluated it using OV differences, and only a few considered both measures. Moreover, most studies only applied virtual surgical planning (VSP) to the intervention group, which could favor the outcomes and create bias. The study by Consorti, Betti, et al. [6] was the only study to apply VSP across both groups, ensuring that their outcomes could be attributed solely to the intervention. However, Gallego-Albertos et al. [8] correctly noted that the use of customized implants and navigation complicates assigning the improved precision to one technology alone. Furthermore, three studies completely lacked a comparison group, which could affect the strength of their findings. Surgical timing, surgeon experience, technique variations, imaging quality, and patient-specific anatomy could influence the outcomes. Lastly, almost all studies, except for Amin et al. [11], utilized retrospective designs, which are more prone to patient selection bias than prospective designs.

In summary, the substantial heterogeneity in the study design, implant type, surgical workflow, and outcome reporting significantly limits the generalizability of the current evidence in terms of orbital floor reconstruction. Although emerging trends suggest that pre-bent patient-specific implants and virtual surgical planning (VSP) enhance operative precision and reduce intraoperative time, the lack of standardized methodologies precludes definitive clinical recommendations. Future research should prioritize multicenter prospective trials with clearly defined inclusion criteria, uniform surgical protocols, and validated outcome measures—particularly for functional metrics such as diplopia resolution, enophthalmos correction, and ocular motility restoration. Comparative studies should also ensure methodological consistency by incorporating VSP in both intervention and control groups to eliminate planning bias. Finally, formal cost-effectiveness analyses are needed to evaluate the economic feasibility and broader clinical adoption of these technologies within varied institutional and healthcare system contexts.

## 5. Conclusions

VSP and 3D printing improve diagnostics and presurgical planning. Pre-bent PSI enables more accurate and time- and cost-efficient surgeries with enhanced functional outcomes. Customized PSIs can achieve high accuracy and functional outcomes but are limited in terms of time efficiency; hence, pre-bent or preformed implants are faster and more cost-effective in clinical workflows.

Intraoperative navigation enhances precision, minimizes complications, and provides real-time feedback, though cost–benefit data are lacking. It can offer comparable accuracy to customized implants when used with preformed implants. Challenges and limitations in the literature include the variability in the outcome measures, the lack of standardized methodologies, the relatively small number of studies for analysis, and the predominance of retrospective studies.

Future research should utilize prospective study designs, standardized outcome measures, and controlled comparison groups to improve the robustness of the findings.

## Figures and Tables

**Table 1:** Search strategy.

PubMed	((“Orbital Fractures” [MeSH Terms] OR “orbital floor” [All Fields] OR “isolated orbital fracture” [All Fields]) AND (“Technology” [MeSH Terms] OR “Therapeutics” [MeSH Terms])) OR ((“Orbital Fractures” [MeSH Terms] OR “isolated orbital fracture” [All Fields] OR “orbital floor” [All Fields]) AND (“Technology” [MeSH Terms] OR “Therapeutics” [MeSH Terms] OR “compar *” [All Fields] OR “analys *” [All Fields]) AND (“Patient specific implant” [All Fields] OR „customized implant” [All Fields] OR “imaging, three dimensional” [MeSH Terms] OR “3D print *” [All Fields] OR “Intraoperative Navigation” [All Fields] OR “hydroxyapatite” [All Fields])) NOT (“paediatric” OR “child *”) AND (y_5[Filter])	135
Scopus	TITLE-ABS-KEY (orbital AND floor AND fracture) AND (TITLE-ABS-KEY (intraoperative AND imaging) OR TITLE-ABS-KEY (intraoperative AND computed AND tomography) OR TITLE-ABS-KEY (intraoperative AND navigation) OR TITLE-ABS-KEY (3d AND planning) OR TITLE-ABS-KEY (three-dimensional AND planning)) AND PUBYEAR > 2018 AND PUBYEAR < 2025 AND (LIMIT-TO (LANGUAGE, “English”))	51
PRIMO	Any field contains orbital floor fracture AND Any field contains Intraoperative Navigation OR Intraoperative Imaging OR three-dimensional print * OR 3D Model OR preformed patient specific mesh OR Any field contains isolated orbital floor AND Any field contains Intraoperative Navigation OR Intraoperative Imaging OR three-dimensional print * OR 3D Model	43

* to capture multiply word forms like analysis, analysed, analyzing; instead of only searching the term “analys”.

**Table 2:** ROBINS-I risk of bias assessment for retrospective studies.

Author (Year)	1	2	3	4	5	6	7
Consorti, Betti et al., (2024) [6]	M	M	L	L	L	M	L
Raveggi et al., (2023) [7]	M	M	L	L	L	M	L
Gallego-Albertos et al., (2020) [8]	M	L	L	L	L	M	L
Probst et al., (2021) [9]	M	M	L	L	L	M	L
Krasovsky et al., (2021) [15]	S	M	L	L	L	M	L
Sigron et al., (2020) [16]	M	L	L	L	L	M	L
Sigron et al., (2021) [17]	M	L	L	L	L	M	L
Consorti, Monarchi et al., (2024) [18]	M	M	L	L	L	M	L

1—Bias due to confounding; 2—bias in the selection of participants; 3—bias in the classification of interventions; 4—bias due to deviations from the intended interventions; 5—bias due to missing data; 6—bias in the measurement of outcomes; 7—bias in the selection of the reported results. L—low; M—moderate; and S—serious risk of bias.

## Data Availability

The data presented in this study are available upon request from the corresponding author.

## Abbreviations

The following abbreviations are used in this manuscript:

VSP	Virtual surgical planning
PSI	Patient-specific implants
OV	Orbital volume
CAD	Computer-aided design
3D	Three-dimensional
CAM	Computer-aided manufacturing
CT	Computer tomography
PICO	Population, intervention, comparison, outcome
PRISMA	Preferred Reporting Items for Systematic reviews and Meta-Analyses
STL	Standard tessellation language

## Appendix A. Data Collection of the Study Results Used for Analysis in the Review

**Author/ Year/Journal**	**Study Design**	**Patients**	**Intervention**	**Comparison**	**Outcome Variables**	**Author Conclusion**
Consorti, Betti, et al. (2024); Journal of Cranio-Maxillo-facial Surgery; [6]	Retrospective	52	VSP + Customized implants + Navigation	VSP + Preformed mesh + Intraoperative Navigation	1. Accuracy (position + OV) 2. Clinical outcomes (diplopia, enophthalmos, ocular motility)	Same accuracy when using VSP and intraoperative navigation, Recommended 3D preformed meshes due to similar clinical outcomes, reduced time, costs benefits
Raveggi et al. (2023); Journal of Cranio-Maxillo-facial Surgery; [7]	Retrospective	73	VSP + Preformed mesh + Navigation		1. Accuracy (Position + Observer classification) 2. Postoperative outcomes (alignment of reconstructed orbital bones, complication rates, patient recovery)	Navigation: Sig. enhanced accuracy, Better alignment and reduced rates of postoperative complications
Gallego-Albertos et al. (2020); Revista Española de Cirugía Oral y Maxilofacial; [8]	Retrospective	35	VSP + 3D Model, pre-bent PSIs + Navigation	Preformed mesh	1. Accuracy (Position + OV), Complications (diplopia, globe position) 2. Reoperation rates	Navigation: Sig. improved positioning, reduced complications, Reduced reintervention rates
Probst et al. (2021); Journal of Clinical Medicine; [9]	Retrospective	27	VSP + Customized implants		1. Accuracy (position) 2. Clinical outcomes (diplopia, revision surgeries), operation time	PSI: Free-hand placement is accurate, No sig. association between the accuracy of PSI placement and unfavourable clinical outcomes
Amin et al. (2024); Journal of oral and Maxillofacial Surgery; [11]	Prospective, Randomized	25	VSP + 3D Model, pre-bent PSIs	Standard mesh	1. Surgery duration 2. Time required for plate insertion and fixation, operating room costs, Accuracy (OV)	PSI: Sig. reduced surgery length, Decreased operating room costs
Krasovsky et al. (2021); Computers in Biology and Medicine; [15]	Retrospective	23	VSP + Customized implants	Preformed mesh	1. Accuracy (position) 2. Early and late complications (diplopia, restricted eye movement, enophthalmos, hyperglobus, exophthalmos)	PSI: Sig. superior accuracy, Sig. fewer complications
Sigron et al. (2020); Journal of Clinical Medicine; [16]	Retrospective	22	VSP + 3D Model, pre-bent PSIs	Standard mesh	1. Accuracy (OV) 2. Surgery duration	PSI: More accurate, sig. different OV, Sig. reduced surgery time
Sigron et al. (2021); Journal of Clinical Medine; [17]	Retrospective	30	VSP + 3D Model, pre-bent PSIs	Standard mesh	1. Clinical outcomes (diplopia, enophthalmos, ocular motility, sensory disturbance) 2. Surgery duration, time between trauma and surgery, time to discharge	PSI: Improvement in functional outcomes, Sig. shorter surgery time, Difference between the 3 surgical metrics not sig.
Consorti, Monarchi, et al. (2024); Life; [18]	Retrospective	26	VSP + Preformed mesh + Navigation		1. Accuracy (Position and OV) 2. Resolution of preoperative diplopia and enophthalmos	Navigation: Precise orbital reconstruction with sig. improved clinical outcomes
